# Estimating limits for natural human embryo mortality

**DOI:** 10.12688/f1000research.9479.1

**Published:** 2016-08-26

**Authors:** Gavin E. Jarvis

**Affiliations:** 1 Department of Physiology, Development and Neuroscience, University of Cambridge, Cambridge, CB2 3EG, UK

**Keywords:** early pregnancy loss, embryo mortality, human chorionic gonadotrophin, fecundability

## Abstract

Natural human embryonic mortality is generally considered to be high. Values of 70% and higher are widely cited. However, it is difficult to determine accurately owing to an absence of direct data quantifying embryo loss between fertilisation and implantation. The best available data for quantifying pregnancy loss come from three published prospective studies (Wilcox, Zinaman and Wang) with daily cycle by cycle monitoring of human chorionic gonadotrophin (hCG) in women attempting to conceive. Declining conception rates cycle by cycle in these studies indicate that a proportion of the study participants were sub-fertile. Hence, estimates of fecundability and pre-implantation embryo mortality obtained from the whole study cohort will inevitably be biased. This new re-analysis of aggregate data from these studies confirms the impression that discrete fertile and sub-fertile sub-cohorts were present. The proportion of sub-fertile women in the three studies was estimated as 28.1% (Wilcox), 22.8% (Zinaman) and 6.0% (Wang). The probability of conceiving an hCG pregnancy (indicating embryo implantation) was, respectively, 43.2%, 38.1% and 46.2% among normally fertile women, and 7.6%, 2.5% and 4.7% among sub-fertile women. Pre-implantation loss is impossible to calculate directly from available data although plausible limits can be estimated. Based on this new analysis and a model for evaluating reproductive success and failure it is proposed that a plausible range for normal human embryo and fetal mortality from fertilisation to birth is 40-60%.

## Introduction

Estimates of natural human embryo mortality have been derived using speculative calculations ^1^, mathematical modelling ^2^, pregnancy surveys ^3^, and a unique collection of surgical material ^4, 5^. Three well-designed studies (henceforth referred to as the Wilcox ^6^, Zinaman ^7^ and Wang ^8^ studies) have shown that approximately two-thirds of menstrual cycles in which elevated human chorionic gonadotrophin (hCG) is detected approximately 1 week after ovulation proceed to a live birth. hCG is produced by the trophoblast cells of the embryo ^9^ and its earliest detection indicates that implantation has commenced ^10– 12^. Hence, these studies provide no direct measure of embryo loss before implantation. The only measure of pre-implantation loss is the “scanty data of Hertig” ^13^ which have generated estimates ^4, 5^ that are “difficult to defend with any precision” ^2^. Estimates of embryo mortality from fertilisation onwards are therefore subject to considerable uncertainty owing to the absence of suitable data for the 5–7 day period between fertilisation and implantation.

Fecundability is the probability of reproductive success per cycle. Compared to other animals, fecundability in humans is low and has been estimated at <35% ^14, 15^. Red deer hinds, by contrast, achieve pregnancy rates of >85% per natural mating ^16^. Clearly, as fecundability increases, the range of plausible values for embryo mortality narrows. Crude estimates of live birth fecundability can be calculated from prospective study data: 19.2% (136 births from 707 cycles ^6^), 18.2% (79 births from 432 cycles ^7^) and 23.9–25.9% (373 births and 31 ongoing pregnancies from 1,561 cycles ^8^). These represent lower limits for fecundability, since optimal conditions for reproductive success were not achieved in every cycle ^17^. However, some published estimates of embryo mortality, *e.g.*, 76% ^2, 18^ and 78% ^1^ can only be reconciled with these data if it is assumed that almost every non-birth cycle in these studies resulted in successful fertilisation and subsequent embryonic or fetal death, an extreme and improbable condition. Higher estimates of embryo mortality, including >85% ^19^ and 90% ^20^, are even less plausible. Furthermore, it is self-evident that not all observed reproductive failure is necessarily due to embryo or fetal mortality: other biological causes include mistimed coitus and failure of fertilisation despite *in vivo* co-localisation of ovum and sperm. Estimates of embryo mortality based on fecundability must take this into account.

The objective of this study is to obtain plausible estimates of fecundability and early human embryo mortality from available published data ^6– 8^. To do this, a simple quantitative framework is proposed to define a successful reproductive cycle. Hence, for a menstrual cycle to conclude with a live infant several distinct biological stages must be completed, each with its own probability ( *π*) of success. These stages (and conditional probabilities) are defined as follows: (1) sexual activity within a cycle resulting in sperm-ovum-co-localisation ( *π _SOC_*); (2) subsequent successful fertilisation ( *π _FERT_*); (3) initiation of implantation approximately 1 week after fertilisation as indicated by increased levels of hCG ( *π _HCG_*); (4) progression to a clinical pregnancy ( *π _CLIN_*): the earliest typical clinical indication is an absent menstrual period approximately 14 days after fertilisation, although definitions of clinical pregnancy vary between studies; (5) survival of a clinical pregnancy to a live birth ( *π _LB_*). It is therefore possible to calculate four different fecundabilities (broadly following Leridon ^21^):

1. Total (All fertilisations):            *FEC _TOT_* = *π _SOC_* × *π _FERT_*
2. Detectable (Implantation):        *FEC _HCG_* = *π _SOC_* × *π _FERT_* × *π _HCG_*
3. Apparent (Clinical):                *FEC _CLIN_* = *π _SOC_* × *π _FERT_* × *π _HCG_* × *π _CLIN_*
4. Effective (Live Birth):            *FEC _LB_* = *π _SOC_* × *π _FERT_* × *π _HCG_* × *π _CLIN_* × *π _LB_*

Quantitative differences between these fecundabilities reflect intrauterine mortality at different developmental stages. Hence, the probability that a fertilised egg will perish prior to implantation is [1 − *π _HCG_*], and prior to clinical recognition is [1 – ( *π _HCG_* × *π _CLIN_*)]. In theory, embryonic mortality may be estimated at all stages although in practice this depends on available data.

In 1969, Barrett & Marshall analysed the relationship between coital patterns and conception and concluded that fecundability increased with coital frequency up to 68% for daily intercourse ^22^. Schwartz’s re-analysis of the same data revealed a similar pattern, although at higher coital frequencies estimated fecundability was lower, at 49% for daily intercourse ^23^. These analyses indicate that failure to conceive at coital frequencies of less than once per day is, in part, due to mistimed coitus and not solely failure of fertilisation and/or embryo mortality. The difference in their estimates of fecundability arises because of key differences between the two analyses. Firstly, Schwartz analysed 2,192 cycles, 294 more than Barrett & Marshall. Secondly, the measures of conception differed: Barrett & Marshall used “absence of menstruation, after ovulation”, approximately 2 weeks after ovulation, whereas for Schwartz conception was “defined as a pregnancy lasting at least 2 months from the last menstrual period”, *i.e*., approximately 6 weeks from the day of ovulation. It is not surprising therefore that Schwartz values were lower since they will not have captured pregnancies that failed between 2 and 6 weeks post-fertilisation. Thirdly, and importantly, Schwartz introduced a new term, ‘cycle viability’, into the analytical model.

Schwartz modelled the probability of conceiving during a cycle ( *i.e*., fecundability, *FEC*) as the product of three conditional probabilities as follows: *FEC* = *P _o_P _f_P _v_*. *P _o_*, *P _f_* and *P _v_* were the probabilities that (i) a fertilisable egg is produced ( *P _o_*), (ii) it is fertilised once produced ( *P _f_*), and (iii) it survives to be detected as a conception ( *P _v_*). *P _f_* was modelled as a function of coital frequency. Cycle viability ( *k*) was defined as *k* = *P _o_P _v_*, and allows for the possibility that optimally-timed coitus would not result in a detected conception. It implies that there is a proportion of cycles that are infertile irrespective of coital activity. Although Schwartz did not explicitly report statistical data demonstrating that the extra parameter ( *k* = 52%) improved the quality of the model, a comparison of the Barrett & Marshall and Schwartz models using the Wilcox study data ^6^ provided compelling statistical evidence to this effect, and concluded that only 37% of cycles were ‘viable’ ^24^.

Since cycle viability ( *k*) includes terms defining reproductive success both before ( *P _o_* = successful ovulation) and after ( *P _v_* = embryo survival) fertilisation, it is not possible to use this term to make direct inferences about early embryo mortality. Nevertheless, Schwartz assumed that *P _o_* = 100%, thereby interpreting all cycle non-viability as a consequence of embryo loss at a rate of 48% during the first 6 weeks after fertilisation. Similar logic applied to the Wilcox study ^24^ would conclude an equivalent estimate of 63% embryo mortality. Schwartz also concluded that *P _f_* = 94% for daily intercourse (0.49/0.52). Hence, Schwartz attributed almost all the observed reproductive inefficiency to embryo mortality and other processes of the reproductive process were, by implication, considered to work almost perfectly. By contrast, referring to fertilisation, Hertig noted that “it seems unlikely that such a complicated process should work perfectly every time” ^5^. It has also been correctly pointed out that preimplantation loss is statistically indistinguishable from other causes of cycle non-viability including male factors ^15^. It seems that this interpretation of reproductive inefficiency has contributed to a widespread impression that early human embryo mortality is very high.

What are the potential explanations for cycle non-viability? Incorporation of a between-couple random effect into the modelling of these data has confirmed that cycle viability is heterogeneous between couples ^15^. A subject-specific random effects modelling approach also resulted in a more consistent cycle by cycle estimate of cycle viability ^25^. These analyses formally demonstrate that within the cohorts of women used in this study, there were individual differences in fecundability. Furthermore, in the Wilcox study, 14 out of 221 women were unable to conceive within 24 months ^6^: this observation alone suggests that a proportion of the study participants were sub-fertile.

Each of the three hCG studies sought to recruit normally fertile, non-contracepting women who intended to conceive. Subjects either had “no known fertility problems” ^6^, or were excluded if they had any “known risk factors for infertility” ^7^ or “had tried unsuccessfully to get pregnant for ≥1 year at any time in the past” ^8^. However, such criteria cannot guarantee complete exclusion of sub-fertile or infertile couples, and in each study pregnancy rates declined in successive cycles as the presumed proportion of sub-fertile women remaining increased. Hence, calculations based on overall aggregate data underestimate fecundability in normally fertile women. Even estimates based on first cycle data are likely to be biased since a proportion of sub-fertile of women would be in the starting cohort. The extent of the bias of such estimates will depend on factors including the heterogeneity of the population and the number of cycles studied.

Estimates for *FEC _HCG_* of 30% ^7^ and 40% ^8^, and for *FEC _CLIN_* of 30% ^8^ and 25% ^6^ probably underestimate the fecundability of reproductively healthy women owing to a mixed fertile/sub-fertile population in these studies. The object of the present analysis was to determine whether the published aggregate data supported this hypothesis and to estimate fecundability for any sub-cohorts identified. The modelling approach is conceptually simple; nevertheless, the results strongly indicate that the hypothesis is true and therefore provide less biased estimates of fecundability for reproductively normal women. These higher estimates of fecundability narrow the range of plausible values for embryo mortality in normal fertile women.

## Methods

**Figure 1.**
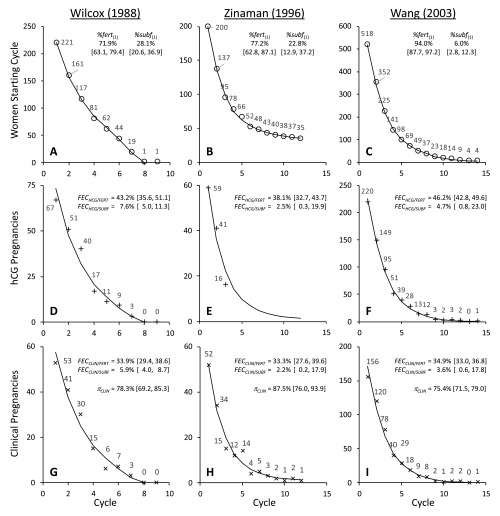
Graphical representation of data and best fit models for Wilcox (A, D, G), Zinaman (B, E, H) and Wang (C, F, I) studies. Each panel shows the data value from the study for each point (○ = women starting cycle; + = hCG pregnancies; × = clinical pregnancies). The line indicates the best fit models as defined in Table 1. Parameter estimates and [95% confidence intervals] from these models are also shown.

Data were obtained from Table 2 of Wilcox ^6^, Table 3 and Figure 1 of Zinaman ^7^ and Table 2 of Wang ^8^ studies. Fourteen women who did not conceive after 24 months were included in the analysis of the Wilcox data (1 reproductive cycle per month was assumed). A subsequent publication reported an extra cycle and an extra hCG pregnancy ^26^; however, it is not clear in which cycle this occurred, and so the original report data ^6^ have been used. In Wilcox and Wang, for each study cycle, the number of (i) women starting each cycle, (ii) hCG pregnancies, and (iii) clinical pregnancies were recorded. The number of women who finished the study without becoming clinically pregnant and the number of women who dropped out at the end of each cycle were also reported. Women who conceived an hCG positive pregnancy but not a clinical pregnancy in a cycle continued in the study. Wilcox reported data for a maximum of nine cycles per subject and Wang for 14. The Zinaman study was similar, except that hCG data were obtained for only the first three study cycles. In the subsequent nine cycles only clinical pregnancy was recorded. Also, only the first pregnancy, whether hCG or clinical was reported.

**Table 1.** Parameter values and statistical output from best fit models ( *Model 0*) of the data from Wilcox (1988), Zinaman (1996) and Wang (2003) studies. Probabilities and percentages were estimated as logits (base 10). Standard errors are shown. Actual probabilities with 95% confidence intervals are reported in Figure 1. Two alternatively parameterised ( *Model 0* & *Model 00*) but statistically identical models were used to obtain standard errors for *FEC _HCG_* and *FEC _CLIN_* since *FEC _CLIN_* = *FEC _HCG_* × *π _CLIN_* ( *ELS* = extended least squares; dof = degrees of freedom.)

Parameter	Wilcox (1988)	Zinaman (1996)	Wang (2003)
*%fert*_(1)_ *FEC _HCG/FERT_* *FEC _CLIN/FERT_* *FEC _HCG/SUBF_* *FEC _CLIN/SUBF_* *π _CLIN_* *σ* *γ*	0.408 ± 0.085 -0.118 ± 0.066 -0.291 ± 0.043 -1.087 ± 0.091 -1.200 ± 0.086 0.558 ± 0.099 0.437 ± 0.090 1.26 ± 0.14	0.529 ± 0.145 -0.211 ± 0.049 -0.301 ± 0.057 -1.598 ± 0.476 -1.657 ± 0.477 0.845 ± 0.165 1.250 ± 0.686 0.47 ± 0.37	1.194 ± 0.167 -0.066 ± 0.029 -0.271 ± 0.018 -1.304 ± 0.383 -1.431 ± 0.378 0.488 ± 0.043 0.870 ± 0.193 0.84 ± 0.13
			
*N* parameters dof *ELS*	27 6 21 52.6707	26 6 20 73.0862	41 6 35 119.209

**Table 2.** Statistical results of hypothesis tests comparing the models shown in Table 1 ( *Model 0*) with alternative models. Degrees of freedom (dof) is the difference in the number of estimated parameters between the models. χ ^2^ is the difference in objective function values ( *ELS*) for the two models. *P* values were calculated using likelihood ratio tests. The models are defined in brackets. H _0_ is the null hypothesis. H _1_ is the alternative hypothesis. NONMEM control files are named according to the study and the model, *e.g.*, Model 0 for the Wang data is WANG0.ctl.

Hypothesis Test	H _0_	H _1_	dof	Wilcox (1988) χ ^2^, *P*	Zinaman (1996) χ ^2^, *P*	Wang (2003) χ ^2^, *P*
1	*FEC _HCG/FERT_* = *FEC _HCG/SUBF_* ( *Model 1*)	*FEC _HCG/FERT_* ≠ *FEC _HCG/SUBF_* ( *Model 0*)	2	54.0, 2 × 10 ^-12^	54.9, 1 × 10 ^-12^	69.5, 8 × 10 ^-16^
2	2 *FEC _HCG_* sub-cohorts ( *Model 0*)	3 *FEC _HCG_* sub-cohorts ( *Model 2*)	2	0.00, 1.00	0.65, 0.72	0.00, 1.00
3	2 *FEC _HCG_* sub-cohorts 1 *π _CLIN_* sub-cohort ( *Model 0*)	3 *FEC _HCG_* sub-cohorts 3 *π _CLIN_* sub-cohorts ( *Model 3*)	4	0.30, 0.99	1.49, 0.83	0.64, 0.96
4	*γ* = 0 ( *Model 4*)	*γ* ≠ 0 ( *Model 0*)	1	34.3, 5 × 10 ^-9^	1.64, 0.20	42.8, 6 × 10 ^-11^

**Table 3.** Estimates of conditional probabilities for different stages of the reproductive process for reproductively normal subjects. Estimates of hCG ( *FEC _HCG_*) and clinical ( *FEC _CLIN_*) fecundabilities and *π _CLIN_* are derived from three hCG pregnancy studies as described in the text. *π _LB_* is calculated from published values in Wilcox ^6^, Zinaman ^7^ and Wang ^8^ study reports. Estimates of fertilised egg loss up to implantation, clinical recognition and birth are provided, based on three scenarios: (i) high implantation probability ( *π _HCG_* = 90%); (ii) equal implantation and fertilisation probabilities ( *π _FERT_* = *π _HCG_*); (iii) high fertilisation probability ( *π _FERT_* = 90%). The probability of sperm-ovum-co-localisation ( *π _SOC_*) was assumed to be 0.80.

Derived Fecundabilities and Conditional Probabilities For Fertile Women	Wilcox (1988)			Zinaman (1996)			Wang (2003)		
*FEC _HCG_* *FEC _CLIN_* *π _CLIN_* *π _LB_*	0.432 0.339 0.783 0.877			0.381 0.333 0.875 0.790			0.462 0.349 0.754 0.871		
% loss from implantation to live birth	31.3			30.9			34.2		
If *π _SOC_* = 0.80, then *π _FERT_* × *π _HCG_* = If *π _FERT_* = *π _HCG_*, then *π _FERT_* = *π _HCG_* = If *π _FERT_* = 0.90, then *π _HCG_* = If *π _HCG_* = 0.90, then *π _FERT_* =	0.540 0.735 0.600			0.476 0.690 0.529			0.578 0.760 0.642		
Estimated losses of fertilised eggs when…	*π _HCG_* = 0.90	*π _FERT_* = *π _HCG_*	*π _FERT_* = 0.90	*π _HCG_* = 0.90	*π _FERT_* = *π _HCG_*	*π _FERT_* = 0.90	*π _HCG_* = 0.90	*π _FERT_* = *π _HCG_*	*π _FERT_* = 0.90
% loss before implantation % loss before clinical recognition % loss before live birth	10.0 29.5 38.2	26.5 42.4 49.5	40.0 53.0 58.7	10.0 21.3 37.8	31.0 39.6 52.3	47.1 53.7 63.4	10.0 32.1 40.8	24.0 42.7 50.0	35.8 51.6 57.8

Observed data were modelled to estimate the following parameters: (1) *%fert*_(1)_ = the percentage of fertile women in the starting cohort; (2) *FEC _HCG_* = the probability of conceiving an hCG pregnancy per cycle; (3) *FEC _CLIN_* the probability of becoming clinically pregnant per cycle. Alternative parameterisation allowed the probability of an hCG pregnancy progressing to a clinical pregnancy ( *π _CLIN_*) to also be determined. The percentage of sub-fertile women in the starting cohort was *%subf*_(1)_ = 100% – *%fert*_(1)_. *FEC _HCG_*, *FEC _CLIN_* and *π _CLIN_* were determined for both fertile and sub-fertile sub-cohorts. The following expressions define the relationship between the parameters and the modelled estimates.

$$N_{FERT(\#)}=N_{\left(\#\right)}\times \%fert_{\left(\#\right)}\;(1)$$

$$N_{SUBF(\#)}=N_{\left(\#\right)}-N_{FERT(\#)}\;(2)$$

$$PREG_{HCG/FERT(\#)}=N_{FERT(\#)}\times FEC_{HCG/FERT}\;(3)$$

$$PREG_{CLIN/FERT(\#)}=N_{FERT(\#)}\times FEC_{HCG/FERT}\times \pi_{CLIN/FERT}\;(4)$$

$$FEC_{CLIN/FERT}=FEC_{HCG/FERT}\times \pi_{CLIN/FERT}\;(5)$$

$$PREG_{CLIN(\#)}=PREG_{CLIN/FERT(\#)}+PREG_{CLIN/SUBF(\#)}\;(6)$$

$$N_{\left(\#+1\right)}=N_{\left(\#\right)}-PREG_{CLIN(\#)}-FIN_{\left(\#\right)}-DROP_{\left(\#\right)}\;(7)$$

$$\%fert_{\left(\#+1\right)}=(N_{FERT(\#)}-PREG_{CLIN/FERT(\#)})÷(N_{\left(\#\right)}-PREG_{CLIN(\#)})\;(8)$$

$$NONPREG_{\left(\#\right)}=[N_{FERT(1)}\times {\left(1-FEC_{CLIN/FERT}\right)}^{\#}]+[N_{SUBF(1)}\times {\left(1-FEC_{CLIN/SUBF}\right)}^{\#}]\;(9)$$

Where: *N*_(#)_ is the number of women starting cycle # (for cycle 1, *N _(_*_1 *)*_ was fixed for each set of study data; Wilcox = 221; Zinaman = 200; Wang = 518); *N _FERT_*_(#)_ and *N _SUBF_*_(#)_ are the modelled number of fertile and sub-fertile women starting cycle #; *PREG _HCG/FERT_*_(#)_ and *PREG _CLIN/FERT_*_(#)_ are predicted numbers of hCG and clinical pregnancies in fertile women in cycle # (and analogously for sub-fertile women); *FIN*_(#)_ is the number of women who finished the study without becoming clinically pregnant in cycle #; *DROP*_(#)_ is the number of women who withdrew from the study at the end of cycle #; *%fert*_(#)_ is the percentage of women starting cycle # who were fertile (and analogously for sub-fertile women); *NONPREG*_(#)_ is the number of non-pregnant women after # cycles ( equation (9) was only used to incorporate 14 non-pregnant women after 24 months into the Wilcox data model). Model expansion to allow three fertility sub-cohorts and contraction to a single fertility sub-cohort enabled hypotheses about parameters and sub-cohorts to be statistically evaluated.

All probabilities and percentages were estimated as logits (base 10). Residual unexplained variance ( *RUV*) was modelled as a function of predicted values ( *PRED*) as follows:

$$RUV=\sigma^{2}\times PRED^{\gamma }\;(10)$$

…where σ is an estimated parameter defining residual error and γ a coefficient defining the relationship between the dependent variables and *PRED*. When γ = 0, the residual model is homoscedastic. When γ = 2, the residual coefficient of variation is a constant.

Data were analysed with NONMEM 7.3.0 (Icon PLC, Dublin, Eire) and implemented using Wings for NONMEM ( http://wfn.sourceforge.net/). Parameters were estimated using a maximum likelihood algorithm (First Order Conditional Estimate with Interaction) and standard errors derived using the inverse Hessian (MATRIX = R). The objective function in NONMEM is the Extended Least Squares ( *ELS*) ^27^. Statistical hypotheses of nested models ( Table 2) were tested using likelihood ratio tests (LRT). Control and data files are available online. Control files are named from the study and the model, e.g., WANG0.ctl is the control file for Model 0 applied to the Wang study data.

## Results

Figure 1 shows the original data values and the fitted models plotted by cycle. Parameter estimates are also shown and output from the models is given in Table 1. These models incorporate discrete fertile and sub-fertile sub-cohorts with differing *FEC _HCG_* but common *π _CLIN_* values. Statistical comparison of alternative models strongly indicated that reducing the dimensionality of the model to a single *FEC _HCG_* value substantially reduced its quality ( Table 2, Hypothesis 1), whereas expanding the model to allow for three different *FEC _HCG_* values did not improve the quality of the model ( Table 2, Hypothesis 2). These statistical results indicate that the data are consistent with bi-modal study populations comprising two distinct fertility sub-cohorts. There was no statistical indication that *π _CLIN_* differed between these sub-cohorts ( Table 2, Hypothesis 3). Evidence for heteroscedasticity in the residual error was strong for the Wilcox and Wang studies, and weak for the Zinaman study ( Table 2, Hypothesis 4).

**Figure 2.**
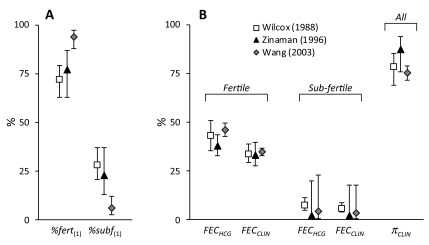
Parameter estimates for fertile and sub-fertile sub-cohorts and associated fecundability values. Values are shown for Wilcox (□), Zinaman (▲) and Wang ( 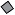) studies. Panel A shows the proportions in the starting cohorts modelled as fertile or sub-fertile ( *%fert*_(1)_ & *%subf*_(1)_). Panel B shows the hCG ( *FEC _HCG_*) and clinical ( *FEC _CLIN_*) fecundabilities and the probability of hCG pregnancies progressing to clinical pregnancies ( *π _CLIN_*). Values are derived from modelled parameter estimates ( Table 1) and error bars indicate 95% confidence intervals.

Figure 2 illustrates the estimated parameter values. Notwithstanding the differences between the studies, there is considerable agreement in the estimates. One noteworthy difference is in the proportion of sub-fertile women. This was low (6.0%) in the Wang study compared to the other two which were approximately 25%. Zinaman *et al.* commented on the high proportion of apparently infertile women in their study despite their efforts during recruitment ^7^. The estimate of 22.8% sub-fertile women is consistent with their estimate of 18% infertility, bearing in mind that sub-fertile women may conceive, albeit with a lower probability. The Wang study was conducted in young Chinese women and had the highest *FEC _HCG/FERT_* (46.2%) and lowest *π _CLIN_* (75.4%) values. This may reflect the Bayesian methodology used to detect hCG positive cycles, the identification of DDT (dichlorodiphenyltrichloroethane), present at unusually high levels in this group ^28^, as a positive predictor of pre-clinical pregnancy loss ^29^, or even a higher incidence of gestational trophoblastic disease in Asian women ^30^.

The analysis also indicates that fewer hCG pregnancies in the Zinaman study (12.5%) failed to progress to clinical recognition, compared to either the Wilcox (21.7%) or Wang (24.6%) studies. This may reflect differences in methodology for detecting hCG, the fact that they made fewer hCG measurements or differences in the definition of clinical pregnancy. Wilcox and Wang defined clinical pregnancy as those that lasted for up to 6 weeks after the last menstrual period ^6, 8, 17, 26^. In Zinaman, clinical pregnancy was determined following serum testing if a woman’s anticipated menses was just one day late ^7^. Hence, the window for pre-clinical embryo loss was approximately 1–4 weeks post-fertilisation for Wilcox and Wang and 1–2 weeks for Zinaman. This different definition of clinical pregnancy would not only contribute to the higher *π _CLIN_* value from Zinaman but also the increased clinical loss of 21.0% compared to 12–13% observed by Wilcox and Wang.

Quantifying the outcome of clinical pregnancies is relatively straightforward. Excluding those lost to follow-up and induced abortions, the probability of a clinical pregnancy progressing to a live birth ( *π _LB_*) was: Wilcox, 87.7% (136/155); Zinaman, 79.0% (79/100); and Wang, 87.1% (373/428). Combining these values with the modelled *π _CLIN_* provides an estimate for embryo loss from implantation to live birth of 31.3% (Wilcox), 30.9% (Zinaman) and 34.2% (Wang) ( Table 3).

Estimating embryo loss prior to hCG detection is less straightforward. For sub-fertile participants, it is impossible to know why they struggled to become pregnant: there are many causes of sub-fertility ^31^. However, for normally fertile women the modelled hCG fecundability values can be used to put limits on fertilisation ( *π _FERT_*) and implantation ( *π _HCG_*) conditional probabilities. As noted above, fecundability is the product of the conditional probabilities of success for each stage of the reproductive cycle. Hence for Wang:

*FEC _HCG_* = *π _SOC_* × *π _FERT_* × *π _HCG_* = 0.462

Since probabilities cannot be greater than 1, the lowest possible value for *π _HCG_* must be 0.462, indicating a maximum possible loss from fertilisation up to implantation in these women of 53.8%. However, it is unlikely that all other probabilities equal 1. Sperm-ovum-co-localisation is dependent on both behavioural and biological factors. As previously noted, the analyses of Barrett & Marshall ^22, 32^ and Schwartz ^23^ show that daily intercourse is more reproductively effective than alternate day intercourse. Hence, at coital frequencies less than once per day, *π _SOC_* must be less than 1. Specifically, a reduction of fecundability from 0.49 with daily to 0.39 for alternate day intercourse ^23^ points towards a reduction in *π _SOC_* of approximately 20%. Volunteers in these hCG studies wished to become pregnant and were undoubtedly aware of the importance of well-timed intercourse. However, they were not required to have daily intercourse and it is likely that in some of the 3,137 cycles intercourse was not always ideally timed. Indeed, in 360/625 cycles in the Wilcox study, intercourse occurred from zero to two times during the 6 days before ovulation, and intercourse occurred on only 40% of the 6 pre-ovulatory days in 625 cycles ^17^. It seems likely therefore that *π _SOC_* and hence fecundability were not maximised in these studies.

Furthermore, not all cycles are ovulatory. Leridon suggested that levels of anovulation lie between 5 and 15% ^33^. Among normal healthy women, the incidence of anovulation ranged from 5.5–12.8% depending on the detection method used ^34^. Therefore, considering behavioural and biological factors together, it seems reasonable to suppose that *π _SOC_* < 1.

It also seems unlikely that either fertilisation or implantation probabilities equal 1. Hence, Table 3 shows derived values for *π _FERT_* and *π _HCG_* assuming that *π _SOC_* = 0.80, and under conditions where: (i) *π _FERT_* = 0.90; (ii) *π _FERT_* = *π _HCG_*; and (iii) *π _HCG_* = 0.90. Based on this analysis, a plausible range for total embryo loss from fertilisation to birth is 40–60%. This is consistent with estimates from both older ^35^ and more recent ^36^ text books. Even with the wide range of mathematically possible outcomes, it is likely that estimates of 90% ^20^, 83% ^37^, 80–85% ^38^, 78% ^1^, 76% ^2^ and 70% ^10, 12^ total human embryonic loss are excessive.

Raw data Wilcox *et al.* study One data file and six control files are provided. The data file is saved as csv. and the control files can be read with any simple text editor. The readme file provides a data legend. Click here for additional data file. Copyright: © 2016 Jarvis GE 2016 Data associated with the article are available under the terms of
the Creative Commons Zero "No rights reserved" data waiver (CC0 1.0
Public domain dedication).

Raw data Zinaman *et al.* study One data file and six control files are provided. The data file is saved as csv. and the control files can be read with any simple text editor. The readme files provides a data legend. Click here for additional data file. Copyright: © 2016 Jarvis GE 2016 Data associated with the article are available under the terms of
the Creative Commons Zero "No rights reserved" data waiver (CC0 1.0
Public domain dedication).

Raw data Wang *et al.* study One data file and six control files are provided. The data file is saved as csv. and the control files can be read with any simple text editor. The readme file provides a data legend. Click here for additional data file. Copyright: © 2016 Jarvis GE 2016 Data associated with the article are available under the terms of
the Creative Commons Zero "No rights reserved" data waiver (CC0 1.0
Public domain dedication).

## Discussion

In 1980, Schwartz wrote that Barrett & Marshall’s estimate of fecundability of 0.68 for daily intercourse “seems to be high”. It implies an absolute maximum limit of embryo mortality of 32%. Schwartz contrasted this with Leridon’s estimate of 44% embryo loss in the first 6 weeks following fertilisation ^3^. However, Leridon’s estimates for early intrauterine mortality are substantially dependent on data and analysis from Hertig ^4, 5^, which are themselves of questionable precision ^2, 13, 39^. Widespread pessimism about human reproductive efficiency may have become a self-fulfilling prophecy in the absence of relevant good quality data.

Nevertheless, Schwartz’s analysis is a useful improvement on that of Barrett & Marshall and points clearly to the presence of infertile or non-viable cycles. The challenge arises in assigning a mechanistic cause for this “non-viability”. Previous reports draw attention to the difficulty of teasing apart distinct components, *e.g*., egg viability versus uterine receptivity ^24^, or male and female factors ^15^, and alternative modelling approaches will yield “different interpretations of the parameters related to cycle viability” ^15^. The advantage of the present models is that the unit of analysis remains the cycle, *i.e*., fecundability, but the heterogeneity of the population is also acknowledged and explicitly incorporated. The model for estimating embryo loss also accommodates other plausible mechanisms for reproductive failure, rather that accrediting all unaccounted reproductive inefficiency to pre-implantation embryo mortality. Although the model does not provide a definitive answer, it does offer plausible limits within which the answer may lie.

The results of this analysis offer a statistically clear picture of bi-modal study populations comprising couples with two discrete levels of fertility. Expanding the model to three levels does not improve this picture and the published data do not support a model of uni-modal, albeit varied, fecundability. Put simply, there was a significant proportion of couples in these studies who were, for unknowable reasons, infertile or clearly sub-fertile. Incorporation of data derived from such couples in calculations to determine normal fecundability will therefore result in biased estimates. By analytically separating the study population into reproductively normal and sub-fertile sub-cohorts, more accurate estimates for normal reproductive function and embryo mortality have been obtained. The analysis presented here cannot be satisfactorily completed owing, in part, to a lack of data on fertilisation success rates *in vivo* ^40, 41^. Consequently, the range for pre-implantation loss, at approximately 10–40%, is wide, although inclusive of Hertig’s pre-implantation loss estimate of 30% ^4, 5^. Despite the imperfections and weaknesses in the available data, it is apparent that plausible values for embryo mortality are considerably less than some figures published in the scientific literature. It is concluded that a plausible range for natural human embryo mortality from fertilisation to live birth in normal healthy women is approximately 40–60%.

## Data availability

The data referenced by this article are under copyright with the following copyright statement: Copyright: © 2016 Jarvis GE

Data associated with the article are available under the terms of the Creative Commons Zero "No rights reserved" data waiver (CC0 1.0 Public domain dedication). 

F1000Research: Dataset 1. Raw data Wilcox *et al.* study, 10.5256/f1000research.9479.d133951 ^42^

F1000Research: Dataset 2. Raw data Zinaman *et al*. study, 10.5256/f1000research.9479.d133952 ^43^

F1000Research: Dataset 3. Raw data Wang *et al.* study, 10.5256/f1000research.9479.d133953 ^44^
