# Supplementary material for: Estimating limits for natural human embryo mortality
Source: F1000Res. 2016 Aug 26;5:2083. [Version 1] doi: 10.12688/f1000research.9479.1 (PMC5142718; doi:10.12688/f1000research.9479.1)
Supplement: Peer review history

# Peer review: Referee response for version 1

**DOI:** 10.5256/f1000research.10209.r16765

**Published:** 2016-10-03

**Authors:** Alan O. Trounson

**Affiliations:** 1 Hudson Institute for Medical Research, Monash University, Clayton, VIC, Australia

This is a well thought out analysis of the data available on human pregnancy wastage. The conclusions are valid from the data explored but the major variant of failed fertilization and very early embryo loss cannot be estimated. Failed fertilization will always be an unknown in such studies but early embryo loss can be estimated from the large amount of IVF data published on embryo survival to the blastocyst stage (day 5-7). However, the vast bulk of this data comes from superovulated patients and this may not represent embryo loss in the natural ovulatory cycle. This data suggests that only 30% of conceptions end up as live babies at delivery (Macklon *et al., *2002)

 Indeed embryonic arrest before day 5 can be attributed to whole chromosome abnormalities in more than half of human embryos (McCoy *et al.,* 2015). The embryonic losses due to mitotic and meiotic support the high embryonic wastage in human reproduction. It is a pity the authors didn’t include this genetic data in support of their hypothesis

I have read this submission. I believe that I have an appropriate level of expertise to confirm that it is of an acceptable scientific standard.

## References

[1] MacklonNSGeraedtsJPFauserBC: Conception to ongoing pregnancy: the 'black box' of early pregnancy loss. *Hum Reprod Update*.8(4) :333-43 1220646810.1093/humupd/8.4.333

[2] McCoyRCDemkoZPRyanABanjevicMHillMSigurjonssonSRabinowitzMPetrovDA: Evidence of Selection against Complex Mitotic-Origin Aneuploidy during Preimplantation Development. *PLoS Genet*.2015;11(10) : 10.1371/journal.pgen.1005601 e1005601 10.1371/journal.pgen.1005601 26491874PMC4619652

# Peer review: Referee response for version 1

**DOI:** 10.5256/f1000research.10209.r15931

**Published:** 2016-09-19

**Authors:** Stephen J. Senn

**Affiliations:** 1 Competence Center for Methodology and Statistics, Luxembourg Institute of Health, Strassen, Luxembourg

This is an interesting and generally well-written article. I am unfamiliar with the field of reproductive physiology and female fertility regulation and so cannot be described as an expert reviewer. However, I do have expertise in the field of statistics and modelling and felt to understand the issues much better having read this article and that is a tribute to its general clarity.

 Nevertheless, at one or two points I felt the clarity could have been improved. The author is not always completely explicit on two points. The first is whether a conditional probability is being estimated (and if so conditional on what) and the second is the precise details of the mixed model being used.

 Since readers will not necessarily be familiar with the software an author uses, and since the more complex the subject the more likely an algorithm will differ between packages, one of the inevitable problems in a field of this complexity are 1) that it is quite likely that readers will not be familiar with some details of implementation and 2) results might differ somewhat from package to package. The author has used NONMEM, a package that is popular in nonlinear mixed effect modelling in pharmacokinetics but less well-known in other fields. This is a limitation of the article. (Not because NONMEM is not a suitable package to use but because it is the only package used.) For example, Makubate and Senn, modelling the effects of cross-over trials in infertility, found some differences depending on whether SAS, GenStat or R were used to  implement what was ostensibly the same model, or indeed program it from scratch using Mathcad and in the field of estimating values below the limit of quantitation Senn, Holford and Hockey got different standard errors using NOMEM compared to SAS, GenStat and R, although such differences are not necessarily inherent to packages but may reflect implementation.

 On a more technical matter, the author has used a discrete mixture which some might regard as being excessively restrictive and a little unrealistic, although the author does claim " *the published data do not support a model of uni-modal, albeit varied, fecundability*". A further issue is that unlike for causal studies, such as clinical trials, the degree to which the subjects studied are representative of a population of interest is important. Lacking knowledge of this particular field and the studies cited I cannot judge whether this condition is satisfied. It seems at least plausible that sub-fertile couples are more likely to be studied than those of average fertility.

 Nevertheless, this seems to be an interesting and valuable exercise in modelling a difficult field.

I have read this submission. I believe that I have an appropriate level of expertise to confirm that it is of an acceptable scientific standard.

## References

[1] MakubateBSennS: Planning and analysis of cross-over trials in infertility. *Stat Med*.2010;29(30) : 10.1002/sim.3981 3203-10 10.1002/sim.3981 21170914

[2] SennSHolfordNHockeyH: The ghosts of departed quantities: approaches to dealing with observations below the limit of quantitation. *Stat Med*.2012;31(30) : 10.1002/sim.5515 4280-95 10.1002/sim.5515 22825800
